# Supplementary material for: Inverted ILM flap technique versus conventional ILM peeling for idiopathic large macular holes: A meta-analysis of randomized controlled trials
Source: PLoS One. 2020 Jul 24;15(7):e0236431. doi: 10.1371/journal.pone.0236431 (PMC7380636; doi:10.1371/journal.pone.0236431)
Supplement: Attachment — Submitted filename: Response to Reviewers.doc [file pone.0236431.s002.doc]

Response to review Comments to the Author

Reviewer #1: The meta-analysis entitled „Inverted ILM flap technique versus conventional ILM peeling for idiopathic large macular holes: a meta-analysis of randomized controlled trials“ is well-conducted the results sound, the information interesting at a first glance. There are, nevertheless, some aspects deserving closer attention:

1. Methodologically, a follow up time of 3 months limits the functional meaning of data, so that functional 3-month data should probably be censored. If not, this has to be pointed on in abstract and text.

Response: Two out of the four studies used in the analysis had a follow-up duration of 3 months. Censoring it would incapacitate this meta-analysis. However, we completely agree with the reviewer that this represents a major limitation and should be clearly communicated to the reader. Therefore, we have added this in the revised version of abstract, in addition to this being pointed out as one of the major limitations in the text (Discussion).

2. The gas tamponade has been discussed but not mentioned in the abstract (i.e. no oil) whereas the dye used for staining was not mentioned though possibly a confounder of results (the toxic ICG is bound in the ILM for long time, whereas trypan blue is not.

Response: Thank you for this valuable suggestion. We have revised the abstract accordingly and the gas tamponade is now mentioned in the text. We agree that ICG could be bound to ILM for a long time and be toxic to the surrounding tissue. None of the studies selected for this meta-analysis have used ICG; 2 studies used Brilliant Blue G, one used Trypan Blue and one used ILM Blue (just another name for Brilliant Blue G Ophthalmic Solution in other markets outside the USA). The dye used for staining has been mentioned in the revised version of text. We have also added the dye used for staining and specified the type of tamponade in the revised version of Table 1.

3. Abstract: explain or remove type 1 closure rates; explain WMD.

Response: We have explained type 1 closure rates and WMD in the revised version of abstract.

4. Introduction: Remove redundant “however” and improve wording, namely of the last paragraph, lines 57-63.

Response: We have removed the redundant “however” and reworded this paragraph in the revised version of the text.

5. M&M: include dye used for staining and specify tamponade, both probably in a table which also includes population demographics to help understanding differences.

Response: We have added the dye used for staining and specify tamponade in the revised version of Table 1. This information was also added in the revised text of the Materials and Methods.

6. Confirm, that MH closure types were reported by all 4 studies included here or re-consider primary outcome definition.

Response: We have revised the primary outcome definition to “anatomic outcome”, and the secondary outcome definition to “visual function outcome”

7. Line 88 and lines 115-7, 187: improve wording.

Response: We have reworded these sentences in the revised version of the text.

8. Lines 134-5, 146-7, 162-3: remove, reiterations.

Response: We have removed these sentences in the revised version of the text.

9. Line 148: remove “with statistically significant” redundancy.

Response: We agree with the reviewers’ comment and have removed the word in the revised version of the text.

10. Line 172: change to …closure of large MHs.

Response: Thank you for pointing this out this discrepancy. We have changed it in the revised version of the manuscript.

11. Line 214: replace conformed by confirmed.

Response: Thank you for pointing out this typo. We have corrected it.

12.Table 1: add reference number.

Response: We have added the reference numbers in the revised version of Table 1.

Manasa: study quality supportable if standard deviation was not determined?

Response:The standard deviation of macular hole diameter was not available in Manasa (2018) study. This limitation was pointed out in the revised text of the Discussion.

Overall rating for Velez-Montoya low because of a small sample size and a short follow up. To be mentioned, data to be used with specific care.

Response: We used the Jadad scale to assess the methodologic qualities of RCTs, using 3 criteria: randomization, masking and participant withdrawals/dropouts. The sample size and follow-up time were not included as criteria in the Jadad scale; therefore Velez-Montoya (2018) ended up with 4 points on the Jadad scale. On the other hand, we completely agree with the reviewer that both small sample size and short length of follow-up are major limitations to this (and similar analyses) and have pointed out these limitations in the relevant paragraph in Discussion.

13. Figures 2-5: Same order of studies as in the table to facilitate reading.

Response: We have reordered the studies in the Figures to match the order in Table 1.

Was the sample size respected for calculation of the OR? Use a decimal or logarithmic scale throughout facilitates reading of the graph.

Response: The sample size is accounted for in the calculation of the OR. The models used to generate the plots in Figure 2 and 3 used the Odds Ratio, while the models used to generate the plots in Figure 4 and 5 used the weighted mean difference. Therebefore, the scales for these figures have to be different.
